# The role of bacteria in oral cancer

**DOI:** 10.4103/0971-5851.76195

**Published:** 2010

**Authors:** Noureen Chocolatewala, Pankaj Chaturvedi, Rushikesh Desale

**Affiliations:** *Department of Oral and Maxillofacial Surgery, M. A. Rangoonwala College of Dental Science and Research Center, Pune, India*; 1 *Department of Head and Neck, Tata Memorial Hospital, Mumbai, India*

**Keywords:** *Bacteria*, *carcinogenesis*, *oral squamous cell carcinoma*

## Abstract

Despite the widening interest in the possible association between bacteria and different stages of cancer development, our knowledge in its relation to oral cancers remains inadequate. The aim of this review article is to derive a better understanding on the role of various micro-organisms in the etiogenesis of oral cancers through all the available data on the pubmed. Different bacteria have been proposed to induce carcinogenesis either through induction of chronic inflammation or by interference, either directly or indirectly, with eukaryotic cell cycle and signaling pathways, or by metabolism of potentially carcinogenic substances like acetaldehyde causing mutagenesis. Studies have shown diversity of isolated bacterial taxa between the oral cancer tissue specimens and the control, with *Exiguobacterium oxidotolerans, Prevotella melaninogenica, Staphylococcus aureus* and *Veillonella parvula* being specific for tumorogenic tissues. Most isolates are saccharolytic and acid tolerant. *Streptococcus anginosus*, commonly linked with esophageal and pharyngeal cancers, is not of significance in oral cancers. Similarly, significant salivary specificity is noted for three bacteria, namely, *Capnocytophaga gingivalis, P. melaninogenica*, and *Streptococcus mitis* in oral cancer patients, making these species salivary markers for the early detection of oral cancers and thus improving the survival rate significantly. Also, such high degree of bacterial specificity in oral cancers has also provoked the designing of new treatment options for cancer prevention by way of vaccine delivery. However, for the success of these steps, a deeper exploration into this subject with a greater understanding is warranted.

## INTRODUCTION

Oral cancers rank sixth amongst the common malignancies globally,[1] with a rising titer of around 40% in developing countries such as southeast Asia.[2] Amongst these, 90% of all oral cancers are squamous cell carcinoma (SCC) originating from the mucosal epithelium.[3]

Although etiology for 70–80% of oral cancers has been majorly linked to betel quid (tobacco) chewing, smoking and alcohol consumption,[4] other factors like genetic susceptibility of the individual, external agents such as dietary factors, may exert their synergistic role in tumorogenesis. Notably, about 15% of oral cancer patients have no known risk factors, and the disease in this population may pursue a particularly aggressive course. This can be attributed to infections involving viruses [Human Papilloma Viruses (HPV) and Epstein Barr Virus (EBV)],[5] fungi like *Candida albicans*[6] and certain bacteria.[7]

The role of viruses in carcinogenesis (by abrogation of p53 and pRb tumor suppressor genes and other cellular proteins involved, with subsequent alteration in the host genome function) is well documented with convincing evidences.[8,9]

Chronic hyperplastic candidosis, a rare oral fungal infection, is associated with the invasion of candidal hyphae into the oral epithelium and known to cause dysplastic changes leading to oral cancers.[6]

Likewise, overwhelming body of study has confirmed the relationship between certain bacteria and cancers. However, there is yet no clear understanding on its mechanism and hence its role remains uncertain. This gap in knowledge makes it impossible to state the exact progression of events by which specific bacteria may cause, colonize or cure cancer.[10,12]

Since not much data are available to support the role of microorganisms in the etiogenesis of oral cancers, we need to further evaluate on this. With this in consideration, we conducted a retrospective systematic review on all the available data from pubmed in order to get a better understanding on the interaction of various type specific microorganisms in oral cancer, and their role in its etiogenesis, if any.

## HISTORY

Several discoveries in microbiological literature since the 19th century have led its way to suggest that bacteria were implicated in all diseases, and hence, the theory of bacterial infections leading to oral cancers was born. Various epidemiological and laboratory-based studies have shown a number of bacterial species to be associated with different cancers [Table 1]. Few such propositions that gained widespread interest were the following revelations

**Table 1:** Various evidence-based cancers associated with specific bacterial etiology

Carcinomas of various regions	Associated pathogen
Gastric carcinoma	*Helicobacter pylori*
Gall bladder carcinoma	*Salmonella typhi*
Cervical carcinoma	*Chlamydia trachomatis*
Lung cancer	*Chlamydia pneumonia*
Intestinal cancer	*Streptococcus bovis*

1. *Helicobacter pylori* infection, known to cause stomach ulcers, can subsequently lead to gastric carcinomas and Mucosa Associated Lymphoid Tissue (MALT).[13–15]
2. Chronic infection with other bacteria, notably *Salmonella typhi*, can also facilitate development of gall bladder cancers.[16–19]
3. *Chlamydia trachomatis* infection has been associated with increased risk for the development of cervical carcinoma.
4. *Chlamydia pneumoniae* may be an etiological source for lung cancer.[20–22]
5. *Streptococcus* bovis mediated bacteremia and endocarditis has been linked with malignancies of colon.[23–26]

Although recently there have been rising evidence to suggest the role of bacteria in cancers, the uneasy understanding and inadequate studies on the complexity of its action in multistage carcinogenesis makes this subject unclarified and warrants a closer study on this issue.

## PROPOSED PARADIGM FOR THE BACTERIAL INVOLVEMENT IN CARCINOGENESIS

**Figure 1:**
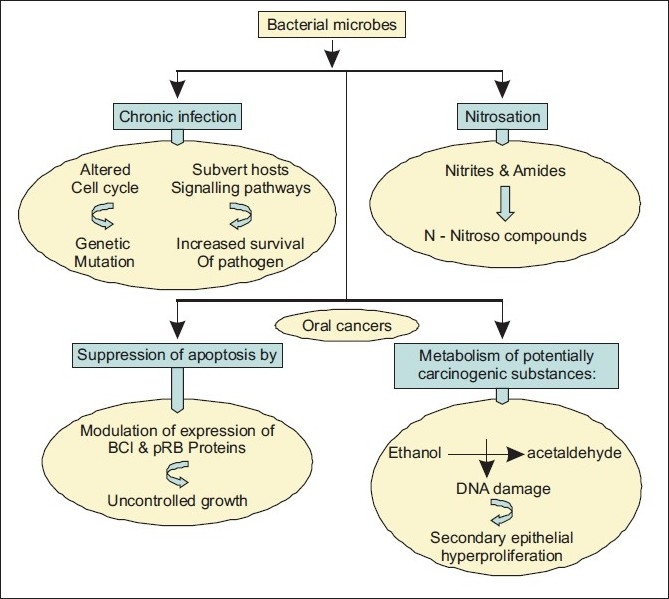
Various proposed paradigms for bacterial role in carcinogenesis

There have been increasing data to confirm that bacterial infections rely upon precise interactions between the pathogens and components of the host cell regulatory systems [Figure 1], which are given below.

1. It has been shown that several bacteria can cause chronic infections or produce toxins that disturb the cell cycle and lead to altered cell growth.[20,21,27]
2. Chronic infections induce cell proliferation and DNA replication through activation of mitogen activated kinase (MAPK) pathways and cyclin D1 and increase the incidence of cell transformation and the rate of tumor development through increased rate of genetic mutation.[28,29]
3. Several infections cause intracellular accumulation of the pathogen, leading to suppression of apoptosis primarily through modulation of the expression of Bcl-2 family proteins or by inactivation of retinoblastoma protein, pRb.[30,31] This strategy provides a niche in which the intracellular pathogen can survive in spite of the attempts of the host immune system to destroy the infected cells by apoptosis. Thus, it allows the partially transformed cells to evade the self-destructive process and progress to a higher level of transformation, ultimately becoming tumorogenic.
4. Many pathogenic bacteria causing chronic infection with intracellular access subvert host cell signaling pathways, enhancing the survival of pathogen.[32] The regulation of these signaling factors is central to the development or inhibition of tumor formation. Such infections can mimic some of the gross effects seen in tumorogenesis, and indeed the precancerous lesion formed in such infections can regress with antibiotic treatment and clearance of bacteria.
5. Another possible mechanism is the metabolism of potentially carcinogenic substances by the bacteria. This is of relevance in the oral cavity, where the pre-existing local microflora may facilitate tumourogenesis by converting ethanol into its carcinogenic derivative, acetaldehyde to levels capable of inducing DNA damage, mutagenesis and secondary hyperproliferation of the epithelium.[33,34] Also, this is evidential from the increased levels of microbial acetaldehyde production in heavy drinkers and smokers, supporting this concept.
6. Microbial carcinogenesis may also involve nitrosation in which microbial cells catalyze the formation of N-nitroso compounds from the precursor’s nitrite and amines, amides or other nitrosatable compounds. Several species of bacteria encompass strains capable of catalyzing nitrosation, in particular, *Escherichia coli*.[35] Also, yeasts and fungi may include nitrosating organisms. This particular nitrosamine appears to be a relevant candidate for the cause of carcinoma, not only of the esophagus but also of other mucosal areas such as the oral cavity.[36]

### Bacteria and oral cancers

For establishing the role of bacteria in the development of oral cancers, it is essential to identify the organisms that prevail in these tumor specimens. This apparent alteration of the oral microbiota in cases of oral squamous cell carcinoma is of particular interest.

This has been highlighted by a study of intraoral carcinomas that demonstrated the difference in the microflora of the tumor tissue with the control sites [Table 2].[11] Although the great diversity of species isolated from a relatively low number of patients’ specimen made it difficult to draw the statistical analysis, a number of interesting trends were apparent.

**Table 2:** Various microorganisms associated with oral cancers

Bacteria isolated from the tumorous specimen	*Exiguobacterium oxidotolerans, Prevotella melaninogenica, Staphylococcus aureus and Veillonella parvula*
Bacteria isolated with the tumor associated saliva sample	*Capnocytophaga gingivalis, Prevotella melaninogenica, Streptococcus mitis*

1. Species detected in the control sites but not in the tumorous specimen were *Moraxella osloensis, Prevotella veroralis* and species of *Actinomyces*.
2. Species isolated from the tumorous sites but not from the control sites were *Exiguobacterium oxidotolerans, Prevotella melaninogenica, Staphylococcus aureus, Veillonella parvula* and species of *Micrococcus*.
3. Majority of the isolates were saccharolytic and acid tolerant, such as yeasts, actinomycetes, bifidobacteria, lactobacilli, streptococci and *Veillonella*. This is in agreement with the fact that the microenvironment of solid tumors is typically hypoxic with low pH, thus favoring the survival of only acid tolerant bacteria.[37,38]

These findings were derived after the specimen surface was decontaminated using betadine solution and phosphate buffered saline washings. The diversity of bacterial taxa in both tumorogenic and control specimens dictates a degree of bacterial specificity and speculates its role in carcinogenic process.

Another study[10] conducted on the biofilms present on the surface of the oral SCC in patients also suggested an increase of the *Veillonella, Fusobacterium, Prevotella, Porphyromonas, Actinomyces* and *Clostridium* (anaerobes), and *Hemophilus*, Enterobacteriaceae and *Streptococcus* spp. (aerobes).

Of particular interest is the implementation of *Streptococcus anginosus* in the process of carcinogenesis, both because of its association with tumor and its ability to induce inflammation. It has been reported that the oral bacterium *S. anginosus* is associated with esophageal, gastric, and pharyngeal cancer tissues.[39,40] A study using highly specific quantification method of real-time polymerase chain reaction (PCR) for *S. anginosus* DNA demonstrated that this bacterium prevailed 10 times more in esophageal cancer tissues than in oral cancer.[41]

A study[42] conducted on 45 OSCC patients showed that six common bacteria – *P. melaninogenica, Capnocytophaga gingivalis, Capnocytophaga ochracea, Eubacterium saburreum, Leptotrichia buccalis and Streptococcus mitis* – were found at significantly higher levels in OSCC patients compared with the controls. Among the 229 cancer-free subjects, the median DNA counts of *C. gingivalis, P. melaninogenica*, and *S. mitis* were 0.25×10^5^/ml of saliva, 0.63×10^5^/ml of saliva, and 0.31×10^5^/ml of saliva, whereas in 45 cancer patients, the median DNA counts of these three bacteria were 3.24×10 ^5^/ml of saliva, 5.62×10 ^5^/ml of saliva, and 1.62×10^5^/ml of saliva, respectively. One possible explanation in this difference in rate could be the change in the cell surface receptors on the epithelium as cancer progresses, enhancing the ability of specific bacteria to colonize.

## FUTURE PROSPECTS

Cancer is commonly defined as the abnormal mass of cells that have inflicted genetic mutation and overcome the various restraints of the normal cell cycle, resulting in its uncontrolled growth. Animal studies have shown that bacterial species present in the blood show preferential seeding at the tumor site. Apparent diversity in the microbial taxa in both tumorous and control tissues suggests some degree of bacterial specificity that calls for further investigation.[10,39–42] Whether the bacteria simply represent secondary colonization or actually participate in carcinogenesis by bacterial–host interactions are ideas that remain inconclusive and need to be further highlighted.

Several pathogenic bacteria causing protracted course of infection can promote or initiate abnormal cell growth by demolishing the host’s immune system or suppressing apoptosis.[44–46] Intracellular pathogens survive by evading the ability of the host to identify them as non-self. Other species secrete toxins that can alter host cell cycles or stimulate the production of inflammatory substances causing DNA damage.[20,21,27–29]

A screening test for oral cancer based on salivary counts of bacterial species is appealing.[39] Currently, saliva is meeting the demand for inexpensive, noninvasive, and easy-to-use diagnostic aid for oral and systemic diseases, and for assessing risk behaviors such as tobacco and alcohol use.

The role of species that colonize tumors could be causal, coincidental or potentially protective. Bacteria bind to and colonize mucosal surfaces in a highly selective manner via a “lock and key” mechanism.[46–49] The specificity of the bacterial species adhering to the tumor surface could be attributed to the presence of their corresponding complementary receptors with lowered immunity, resulting in the shift of oral microflora toward the unfavorable end. The other logical cause would be the irregularity of the lesion surface favoring microbial retention, especially of the anaerobes.

A study[50] on *Streptococcus sanguis*, a common oral inhabitant, demonstrated that this bacterium adhered only to those exfoliated normal Human Buccal Epithelial Cells (HBEC) that contained surface sialic acid residues. Desialylation of HBEC invariably abolishes adhesion of *S. sanguis* to these epithelial cells as seen in buccal carcinoma cell line. This suggests that changes in the surface receptors do occur in the buccal carcinoma.

Currently, the best method of detecting oral cancer is an annual examination of the mouth, head and neck. So far, however, general population screening has not been shown to reduce the incidence or mortality of oral cancer, and so there is a demand for an inexpensive, noninvasive and easy-to-use diagnostic test for oral cancer.[51]

As saliva is shown to have a microbial profile similar to that of the soft tissues, shifts in the oral soft tissue microbiota in cancers appear likely to affect salivary levels as well. Also, concomitant presence of periodontal infections or a smoking habit does not affect salivary and soft tissue colonization.

With significant salivary specificity toward three bacteria, namely, *C. gingivalis, P. melaninogenica*, and *S. mitis, as* seen in oral cancers,[42] it may be ideal not only in diagnosing the presence of a malignancy but also in executing the appropriate therapy, thus forming the signature of oral cancers. These findings could form the basis of the development of a straightforward saliva test for the diagnosis of oral cancer. If oral cancer is detected in its early stages, 5-year survival rates would dramatically improve to 80–90%.[42]

On the above basis, some investigators have recently come up with designing new treatments that stimulate the immune system through attenuated bacterial vaccines to recognize and target the lesion by safe and effective delivery of plasmids encoding tumor self-antigens. Cancer vaccines, although promising in treatment and prevention of certain cancer recurrence, present significant challenges in determining the most effective bacterial strains, addressing safety issues and the problem of overcoming the peripheral T cell tolerance against tumor self-antigens.[52–57]

**Figure 2:**
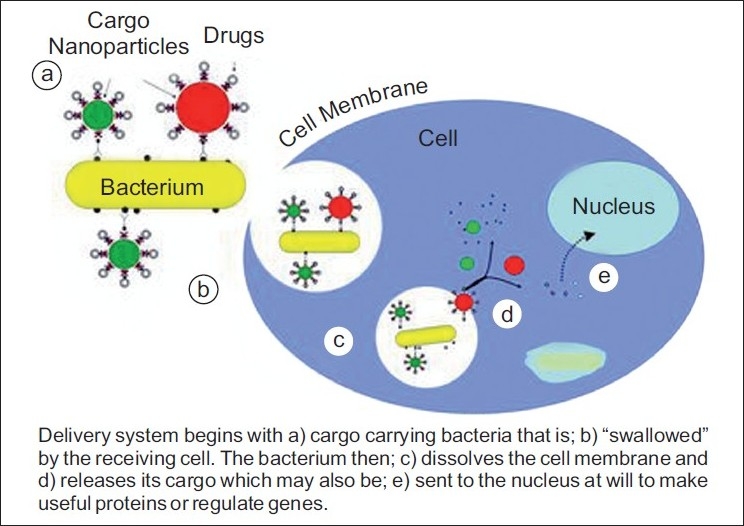
Bacteria laden with “Smart polystyrene nanoparticles” which can carry genes, drugs, nanosensors or other cargo into the interior of host cells for early diagnosis and treatment of oral cancers

Recently, bacteria laden with “smart polystyrene nanoparticles,” which can carry genes, drugs, nanosensors or other cargo into the interior of host cells, are being used to precisely position cargo inside the cells for the early diagnosis and treatment of cancer and other diseases[58] [Figure 2].

## CONCLUSIONS

To summarize, recent research has enlightened us with wider range of information regarding the bacterial mechanisms used to cause, colonize or cure cancer. However, yet many doubts remain untouched. These are: Is it the microbial infections that initiate cancer, or is it the pre-existing cancer that lowers the host’s immunity facilitating secondary microbial colonization? Can the highly site-specific colonization of certain bacteria be of any value in its diagnosis or treatment? Could attenuated bacteria be used in vaccines to modulate host’s immunity against cancer? This calls for further exploration on this subject, which would clear our understanding of the role of the microbial detection, not only in prevention or early diagnosis of oral cancers but also in providing an effective treatment and improving the survival.
